# Ion and Molecule Transport in Membrane Systems 3.0 and 4.0

**DOI:** 10.3390/ijms24098211

**Published:** 2023-05-04

**Authors:** Victor Nikonenko, Natalia Pismenskaya

**Affiliations:** Membrane Institute, Kuban State University, 350040 Krasnodar, Russia

This book is a collection of papers published in the 3rd and 4th Special Issues of the International Journal of Molecular Sciences under the standard title, “Ion and Molecule Transport in Membrane Systems”. The book extends the series that began with the 1st [1] and 2nd [2] Special Issues. The primary focus of this Special Issue is to bring together papers describing ion and molecule transportation in biological or artificial membrane systems.

**Biological Membrane Systems**

The paper by N.-P. Foo et al. [3] investigates how midazolam affects the K^+^ current through cell membranes. The study demonstrated that midazolam suppressed the amplitude of delayed-rectifier K^+^ current. The results considered that midazolam could affect lymphocyte immune functions. Langó et al. [4] developed an effective experimental method for accurately and comprehensively characterizing individual cells’ surface proteins. Such proteins play a crucial role in several critical cellular processes, and identifying surface-associated protein segments has broad applications for molecular biology. Efimova et al. studied the influence of chromone-containing allylmorpholines on ion channels formed by pore-forming antibiotics in lipid membranes [5]. This effect was correlated with allylmorpholine’s ability to affect membrane boundary potentials and lipid-packing stress. 

**Ion and molecule transport in membranes studied by NMR**

The self-organization of fullerene derivatives in solutions and biological cells was studied by Avilova et al. [6] using pulsed gradient nuclear magnetic resonance (NMR). This investigation contributes to a more comprehensive understanding of the mechanisms behind the aggregation of fullerene-derived molecules; it provides the size and stability of associates. This study’s focus is enhanced by the fact that fullerene derivatives are known for their pronounced anticancer and antiviral effects, as well as their antibacterial properties. The following paper [7] is a review prepared by Dr. V. Volkov and colleagues on the long-term results obtained in the field of NMR. This review contains comprehensive information on ion and molecular transport in ion-exchange resins and membranes, such as Nafion, MF-4SK, and MK-40. Self-diffusion coefficients of protons and Li^+^, Na^+^, and Cs^+^ cations are reported along with the ionic conductivity data. The transport channel morphology, ionic hydration, and charge site formation are also discussed. The applications of various NMR modes, high-resolution NMR, solid-state NMR, NMR relaxation, and pulsed field gradient NMR techniques are explored. 

**Artificial Membranes: Gas Molecule Transport**

The separation of pairs of gas molecules (He/N_2_ and O_2_/N_2_) and a methanol(MeOH)–cyclohexane(CH) mixtures using a Ultem^®^ polyetherimide (PEI) membrane with an addition of the perovskite oxide La_0.85_Yb_0.15_AlO_3_ (LYA) was studied by Pulyalina et al. [8]. They found that the selective separation of the gas pairs increased with the growth of LYA content in the membrane. The separation of the MeOH–CH mixture was effective due to the high sorption of MeOH in the PEI/LYA membrane.

The paper by Petriev et al. [9] is devoted to enhancing the performance of composite Nb-based membranes through hydrogen purification via diffusion. Modifications of gas diffusion PdCu–Nb–PdCu membranes with a nanostructured crystalline coating were obtained. It was found that the flux of pure hydrogen through the modified membranes was 1.73 times higher than through the non-modified composite membranes at 300 °C. The mechanisms of the hydrogen flux enhancement due to the modification are discussed. Such high fluxes must be obtained at relatively low temperatures, which, along with cost-efficient niobium-based membranes, provides a promising approach for the economical production of pure hydrogen.

**Artificial Membranes: Ion and Molecule Separation**

The dehydration of ethanol–water mixtures in the broad concentration range was investigated in a pervaporation process studied by Burts et al. [10]. The authors applied thin film composite (TFC) membranes with a polyvinyl alcohol (PVA) selective layer. The effect of modification of the PVA layer with aluminosilicate (Al_2_O_3_·SiO_2_) nanoparticles and PVA-Al_2_O_3_·SiO_2_/polyacrylonitrile (PAN) thin film nanocomposite (TFN) membranes was investigated. The new PVA-Al_2_O_3_·SiO_2_/PAN TFN membranes were found to be more stable in the ethanol dehydration process compared to the reference membrane.

Issues of ion separation in various membrane systems were studied in [11,12,13,14,15].

The simultaneous recovery and concentration of 1-Ethyl-3-methylimidazolium chloride ([Emim]Cl) ionic liquid were performed using electrodialysis by Babilas et al. [11]. Heterogeneous ion exchange membranes were also applied. The effects of varying the [Emim]Cl concentration, applied voltage, linear flow velocity, and the dilute-to-concentrate volume ratio were investigated, leading to the discovery of optimized operational parameters. Another electrodialysis process using a microfluidic system with ion-exchange membranes was studied by Tichý and Slouka [12] where the separation performance was tested by desalting a model KCl solution spiked with fluorescein to directly observe desalination. It was demonstrated, both visually and by measuring the output solution conductivity, that the system can work in three operation modes as follows: continuous desalination, desalination by accumulation, and unsuccessful desalination. The possibility of independent control of different parameters and visualization encourage the consideration of the proposed system as a versatile platform for investigating the electrodialysis process. The separation of hydrochloric acid and Zn^2+^, Ni^2+^, Cr^3+^, and Fe^2+^ salts was studied by Merkel et al. [13] using a spiral-wound diffusion dialysis module. It was established that this process recovers 68% of free HCl from the spent pickling solution contaminated with heavy-metal-ion salts. The effect of different input parameters was investigated. It was shown that diffusion dialysis is an effective and economical method for the treatment of spent acids. The separation of another industrial effluent, that of fermentation broths, was studied by Tomczak and Gryta [14]. The authors applied reverse osmosis (RO), a membrane process, more suitable for this aim. They established that the retention of carboxylic acids increases with increasing molecular weight; the following order was found: succinic acid > lactic acid > acetic acid > formic acid. Pismenskaya et al. [15] looked for ways to enhance the separation performance of weak polybasic acid salts using electrodialysis. The authors discovered that electroconvection makes a significant contribution to the increase in the mass transfer rate, although this contribution is less significant than in the case of electrodialysis of strong electrolytes. This is because a higher generation of H^+^ ions is released during the dissociation of singly charged acid anions at the membrane’s surface.

**Experiment and Mathematical Modeling of Ion and Molecule Transport Processes**

Filippov and Shkirskaya theoretically and experimentally studied osmotic and electroosmotic water transports [16]. The theoretical study is based on the well-known cell model [17] of a charged membrane involving the principles of irreversible thermodynamics. Exact and approximate analytical formulas for calculating the membrane osmotic and electroosmotic permeability are presented. The theoretical results are verified using experimental data for a cation exchange membrane.

Skolotneva et al. [18] studied the transport of ammonium cations through anion-exchange membranes, comparing them with the transport of K^+^ cation. Even though the mobility of both cations in a solution is narrow, the diffusion flux of ammonium through an anion-exchange membrane is significantly higher than that of potassium. The reason is the additional transport of NH_3_ molecules, along with the ammonium cations, since a part of the membrane consists of NH_4_^+^ cations which are transformed into NH_3_ molecules. The authors developed a mathematical model describing the transport of ammonium complicated by a parallel proton-exchange reaction. The comparison of the simulation with the experiment shows a satisfactory agreement. The following three papers [19,20,21] also presented a mathematical description of ion transport in membrane systems. Gorobchenko et al. [19] developed a new mathematical model to describe the competitive transport of cations through a bilayer membrane. The substrate layer was a cation exchange, and a thin surface layer was an anion exchange. The anion-exchange layer has a higher resistance for cation transport than for anion transport; moreover, the resistance for divalent cations is much higher than for those that are monovalent. This explains why such bilayer membranes have selective permeability for monovalent cations. The model predicts that the permselectivity coefficient vs. current density dependence has an upper limit. This dependence is explained by a change in the membrane layer, which controls the rate of ion transport. Another paper reporting the results of the mathematical simulation of ion transport in an anion-exchange membrane modified with a perfluorosulfonated ionomer is published by Kozmai et al. [20]. The idea of the modification was to “clog” the macropores of a commercial membrane and thereby block the transport of co-ions through the membrane. After such a modification, only the highly selective microporous gel phase of the membrane was available for ion transport. The modification allowed reducing the co-ion transport number from 0.11 to 0.02. A new version of the known microheterogeneous model [21] was developed to describe the change of membrane characteristics caused by its modification.

The transport phenomena occurring in reactive electrochemical membranes during the anodic oxidation of organic compounds are investigated by Mareev et al. [22]. The mathematical model developed by the authors examines how the formation of gas bubbles that are generated by electrochemical reactions inside the membrane pores affects the performance of the anodic oxidation of organic compounds, such as paracetamol.

An interesting phenomenon of autowaves in a magnetic fluid was studied experimentally and theoretically by Chekanov and Kovalenko [23]. Magnetic fluids are a colloidal system, like a liquid membrane. They involve ferromagnetic nanoparticles suspended in a carrier fluid’s dispersion medium (for example, kerosene). The system of Nernst–Planck–Poisson and Navier–Stokes equations were applied to describe the dependence of the frequency of concentration fluctuations on the value of a steady-state voltage applied between two electrodes.

A review of the ion and water transport in ion-exchange membranes applied in power generation systems is published by Mareev et al. [24]. The review provides guidelines for modeling the ion and water transport, describing the main structural elements of such membranes and their influence on transport, and considers the features determined by the application area. Significant attention is paid to models with the greatest impact and are most frequently used in the literature.

**In Conclusion**

The original articles and reviews, overviewed in this editorial, provide novel insights into membranes’ ion and molecule transport mechanisms. The readers can also find applied aspects and understand the practical problems that can be solved with the help of membranes. The guest editors would like to thank all authors for their excellent contributions. Having collected the papers published in the Special Issues mentioned above, this book will be an asset to the community of researchers and engineers working in the biological and artificial membranes field.
